# A Perspective on the Characterization of Early Neural Progenitor Cell-Derived Extracellular Vesicles for Targeted Delivery to Neuroblastoma Cells

**DOI:** 10.1007/s11064-024-04165-1

**Published:** 2024-06-05

**Authors:** Oğuz Kaan Kırbaş, Batuhan Turhan Bozkurt, Melis Rahime Yıldırım, Pakize Neslihan Taşlı, Hüseyin Abdik, Fikrettin Şahin, Ezgi Avşar Abdik

**Affiliations:** 1 Department of Genetics and Bioengineering, Faculty of Engineering and Architecture, Yeditepe University, Istanbul, 34755 Turkey; 2 Department of Molecular Biology and Genetics, Faculty of Engineering and Natural Sciences, İstanbul Sabahattin Zaim University, Istanbul, 34303 Turkey; 3 Department of Genomics, Faculty of Aquatic Sciences, Istanbul University, Istanbul, 34134 Turkey

**Keywords:** Extracellular vesicles, Human neonatal dermal fibroblasts, Neurogenic differentiation, Targeted delivery

## Abstract

As an element of the cellular signaling systems, extracellular vesicles (EVs) exhibit many desirable traits for usage as targeted delivery vehicles. When administered, EVs cause little to no toxic or immune response, stay in circulation for longer periods compared to synthetic carriers, preferentially accumulate in tissues that are the same or similar to their cell-of-origin and can pass through the blood-brain barrier. Combined, these traits make neural EVs a particularly promising tool for delivering drugs to the brain. This study aims to combine tissue and EVs engineering to prepare neural differentiated cells derived EVs that exhibit neural properties, to develop an effective, tissue-homing drug and gene delivery platform for the brain. Early neural differentiated cell-derived EVs were produced with neural characteristics from neural differentiated human neonatal dermal fibroblasts. The EVs carried key neural proteins such as Nestin, Sox2 and Doublecortin. The cellular uptake of early neural differentiated cell-derived EVs was higher compared to non-neural EVs during in vitro uptake assays on neuroblastoma cells. Moreover, eND-EVs were significantly decreased the viability of neuroblastoma cells. In conclusion, this study revealed that early neural differentiated cell-derived EVs have potential as a promising drug carrier for the treatment of various neural disorders.

**Graphical Abstract:**

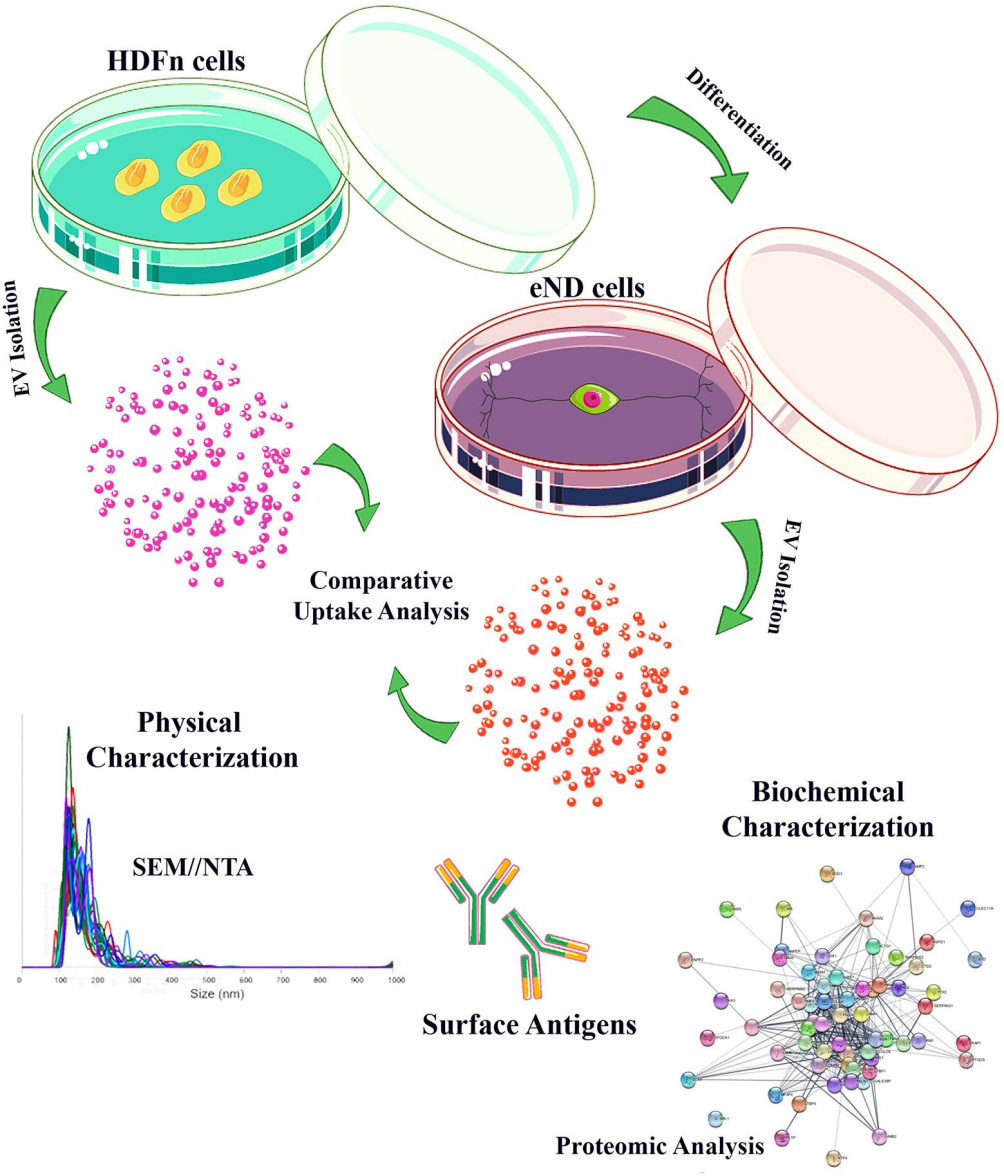

**Supplementary Information:**

The online version contains supplementary material available at 10.1007/s11064-024-04165-1.

## Introduction

Modern drug delivery methods release therapeutics at a specific site of action at a controlled rate. Traditionally, synthetic drug carriers that are engineered to have site-specific and controlled release are used for such applications. However, EVs, lipid-bilayer vesicles secreted from all cell types, could be a promising alternative to such synthetic drug carriers. EVs have become the subject of numerous scientific studies due to the wide range of research opportunities and medical applications they offer. These nano-sized cargo molecules are ubiquitous in our bodies, carrying out crucial functions as a part of cellular signaling pathways. EVs allow an individual cell to affect numerous neighbors simultaneously, which can form and alter the cellular microenvironment, act as an alarm in situations such as tissue damage or immune response, and maintain homeostasis [1].

EVs accomplish these tasks by inducing biological responses in the cells they interact with. The nature of these responses depends on the physiological properties of the cell when it secretes the EV; both the permanent characteristics of the cell and the more transient, at-the-moment micro-environmental effects translate to the final properties of the secreted EV [2]. Therefore, it is possible to produce EVs that can induce the desired effect on recipient cells by modifying the nature of the EV-secreting cell [3].

The innate traits of EVs make them an ideal drug delivery vehicle. Non-autologous or xeno-EVs do not raise an immune response and show little to no toxicity when administered to patients [4], have a longer half-life in circulation compared to synthetic vesicles [5], and avoid lysogenic pathways during their cellular uptake, protecting their cargo [6]. Finally, the surface markers of EVs control their biodistribution in vivo, where they concentrate on tissues similar to their origin [7], or at sites undergoing specific stimuli, such as wounds [8].

The homing ability of EVs is a crucial advantage over synthetic drug carriers, which require complex and expensive surface modifications for the same capacity. Furthermore, modifying an artificial vesicle towards one feature might change another – for instance, increasing the biodegradability of a nanoparticle to reduce its toxicity would also reduce its circulation time [9]. As the composition of EV surface markers depends on the secreting cell, EVs isolated from cells identical or similar to the target tissue naturally carry the necessary targeting elements. Besides, produced by endogenous cellular machinery, their high biocompatibility and low immunogenicity make EVs more efficient and advantageous than synthetic carriers [10]. On the other hand, there are some challenges to overcome in the future clinical implications of EVs. A significant challenge is that EVs consist of heterogeneous vesicle populations. Besides, the scalability and standardization of EV production also pose challenges for clinical studies [11]. Despite all the limitations, these challenges are getting closer to being solved day by day. In combination with the rest of the advantageous traits, the homing ability makes EVs a promising candidate for targeted drug delivery [7, 8].

However, target tissues or cells may not also be suitable for therapeutic EV production. For example, targeting extracranial solid tumor neuroblastoma has several problems. EVs of disease cells are unsuitable to be drug carriers due to their detrimental effects, such as immune suppression [12], or induction of metastasis [13]. While healthy cells of similar origin can be used instead, neurons are hard to acquire and cannot be cultured in vitro conditions for extended periods. Therefore, to be used in studies related to neural cell models, it is known that mesenchymal stem cells or neonatal dermal fibroblasts are successfully differentiated to neuron-like cells. In these studies, it was seen that cell is differentiated by the modulation of particular signaling pathways due to using small molecules. In some studies, cells were differentiated by the inhibition of GSK-3β and SMAD signaling pathways, while in others, cAMP and BMP signaling pathways were used [14, 15]. Moreover, in some studies, EVs of neural origins were obtained from the conditioned media of neurally differentiated mesenchymal stem cells. Naturally, these cellular changes are also reflected in the properties of EVs. A study reported that cyclin D1 protein was enriched in the content of EVs derived from neural differentiated cells. Thus, it was discovered that these EVs can induce neural differentiation [16, 17].

In the study, it is aimed to characterize eND-EVs with a focus on assessing their acquisition of some neuronal features. Therefore, we employed an alternative approach to producing EVs capable of targeting neural cells and diseases. By differentiating HDFn cells into neural progenitors, we produced cells with neural characteristics that are capable of proliferation and extended in vitro culture, suitable for continuous EV production, producing EVs with neural surface proteins.

## Materials and Methods

### Cell Culture Conditions

Human neonatal fibroblast cell line (#HDFn, ATCC® PCS-201-010™) were used for differentiation and also EV source in the study. HDFn and SH-SY5Y (ATCC®, CRL-2266™) cells were cultured in Dulbecco’s Modified Eagle’s Medium (DMEM, #41966-029, Invitrogen, Gibco, UK) supplemented with 10% Fetal Bovine Serum (FBS, #10500-064, Invitrogen, Gibco, UK) and 1% Penicillin/Streptomycin/ Amphotericin (PSA, Invitrogen, Gibco, UK. he incubation condition of the cells was at 37 °C in a humidified atmosphere with 5% CO_2_.

### Neurogenic Induction

HDFn cells were seeded on cell basement membrane **(**ATCC®, ACS-3035™) coated 6-well cell culture dishes at a density of 5000 cells/cm^2^ [18]. For the induction step of differentiation, cells were treated with an induction medium for six days. Early induction medium was composed of Neurobasal medium containing 20ng/mL bFGF, 20ng/mL EGF, 1% B27 supplement, 1% ITS (insulin, transferrin, and selenium), 10% Glutamine, and 1% PSA, and the medium of cells was refreshed every two days. For the differentiation stage, the differentiation protocol was applied by changing the differentiation medium every two days with a Neurobasal medium containing 20ng/mL EGF, 20ng/mL bFGF, 20ng/mL NGF, 20ng/mL NT4, 20ng/mL BDNF, 1X Glutamax for the next six days (Table 1).

**Table 1:** Medium formulations required for early progenitor neural differentiation of HDFn cells

Days 1–6 Early Induction Medium		Days 7–13 Differentiation Medium	
bFGF	20 ng/mL	bFGF	20 ng/mL
EGF	20 ng/mL	EGF	20 ng/mL
B27 supplement	1%	NGF	20 ng/mL
ITS	1%	NT4	20 ng/mL
Glutamine	1%	BDNF	20 ng/mL
PSA	1%	Glutamax	1X

### Cresyl Violet Staining

HDFn cells were stained with Cresyl Violet stain before and after the differentiation process. The medium was removed from the culture wells, and cells were washed thrice with 1X PBS. Cells were then fixed by adding 500µL of 4% paraformaldehyde for 10 min at room temperature. Fixative was then washed away thrice with 1X PBS. 1 ml of 0.4% cresyl violet stain was added to each well, and wells were stained for 30 min at room temperature. Any excess dyes were removed by washing thrice with 1X PBS, and cells were imaged under Axiovert A1 light microscope. At least three pictures were acquired from each well.

### Relative real-time Reverse Transcription Polymerase Chain Reaction

To determine the alteration of gene expressions of HDFn cells during the neural progenitor differentiation procedure, relative real-time RT-PCR analysis was performed. In this respect, the alterations of early neuronal markers in differentiated cells were analyzed. The cells collected on the last day of the neural progenitor differentiation were first isolated with the NucleoSpin RNA (Macherey-Nagel) kit. Afterward, the purity of isolated RNA was analyzed by NanoDrop 2000 (Thermo Scientific), and synthesis of cDNAs was performed with QuantiTect Reverse Transcription kit (Qiagen). cDNAs used in relative qPCR analysis were completed with primers (Table 2) and SYBR up to 10 µl, and the reactions were propagated in CFX96 Touch Real-Time PCR Detection System (Bio-Rad, USA). In the analysis of results, the 18 S rRNA gene was used for the normalization procedure.

**Table 2:** Primer sequences used in relative real-time RT-PCR analysis

Gene name	Sequences (5’-3’)		
	Forward	Reverse	
Doublecortin	CCAAGACGCAAACGGAACCT	AATCACCAAGCGAGTCCGAG	
Nestin	CGCACCTCAAGATGTCCCTC	CAGCTTGGGGTCCTGAAAGC	
SOX2	CAGGAGTTGTCAAGGCAGAGA	CCGCCGCCGATGATTGTTAT	
CXCR4	GCATGACGGACAAGTACAGGCT	AAAGTACCAGTTTGCCACGGC	
ACTA2	CCCTGAAGTACCCGATAGAAC	GGCAACACGAAGCTCATT	
CD90	ATGAACCTGGCCATCAGCA	GTGTGCTCAGGCACCCC	
18 S rRNA	CGGCTACCACATCCAAGGAA	GCTGGAATTACCGCGGCT	

SOX2 SRY-related HMG-box, CXCR4 C-X-C chemokine receptor type 4, ACTA2 smooth muscle alpha actin

### Flow Cytometry Analysis of Neural Lineage Markers

Flow cytometry analysis was performed to examine the changes in the neural protein expressions of neurogenic differentiated cells. Accordingly, at the end of the differentiation process, cells were harvested and washed with PBS solution. Then, the cells were fixed and permeabilized by incubating with fixation/permeabilization solution for 30 min at 4 °C. After washing the cells again, they were blocked by incubating with 1% BSA-containing PBS solution for 30 min at 4 °C. Subsequently, the cells were incubated overnight with 1:100 diluted solutions of Sox-1, CD-44, Doublecortin, Nestin, Ki-67, and GFAP antibodies from the Human Neural Lineage Analysis Kit (561,526, BD, USA). Eventually, the cells were washed twice with PBS, and the flow cytometry analysis was performed with a BD FACSCalibur Flow Cytometer instrument.

### Medium Collection

Differentiated cells were cultured in T-150 cell culture flasks with a differentiation medium. Cells were cultured at a confluency of 60–80%, and the medium was collected every other day for EV isolation. The collected medium was stored at -80 ℃ for further studies.

### Extracellular Vesicles Isolation

Early neural differentiated cell derived-extracellular vesicles (eND-EVs) were isolated from the collected culture medium using an aqueous two-phase system (ATPS) isolation method [19]. In the ATPS method, Polyethylene glycol (PEG) and Dextran were used as phase-forming polymers because they were known to have no effects on cells [20]. Briefly, cells and cellular debris were removed from the medium by centrifugation twice, at 300 g and 2000 g for 10 min. Medium-sized EVs were removed by centrifuging the media at 10,000 g for 10 min, followed by a 0.22 μm filtration. The prepared medium was combined with the ATPS isolation solutions in 50 ml falcon tubes at a 1:1 ratio, which consists of 3,35 w/v PEG (Sigma, #81,310) and 1,65 w/v Dextran (Sigma, #81,392) in 1X PBS. Washing solutions were also prepared during this time by diluting the isolation solution 1:1 with 1X PBS. The washing solutions and the samples were centrifuged at 1000 g for 10 min to form the phases. 80% of the total volume was discarded from the top of the samples and replaced with 80% of the top volume from a washing solution. Samples were centrifuged and washed again, after which their top volumes were discarded, and the bottom phases containing the EVs were collected and stored at -80^o^C for further experiments.

### Nanoparticle Tracking Analysis (NTA)

Size distribution, concentration, and density of specialized EVs were measured using nanoparticle tracking analysis (Nanosight NS300, Malvern Instruments). Samples were diluted to fit the concentrations suggested by the manufacturer. The video capture was done with the low-volume sample chamber for 15 s at camera level 16. The chamber was flushed with distilled water between each capture. A total of 30 captures were taken for each sample [21]. Data analysis was done using NTA software version 3.4.

### Scanning electron Microscopy (SEM)

Electron micrographs were taken for the morphological analysis of EVs. Samples were diluted 1:10 times with 1X PBS and partially dried on carbon disks. Measurements were taken under 100-pascal pressure with a 20 kV charge. Electron micrographs were taken using FEI-SEM Quanta 250 FEG Environmental SEM.

### Simple Western Analysis by Capillary Electrophoresis

Protein expression profiles of isolated EVs were shown by capillary western blot (Wes, Protein Simple; San Jose, CA, USA). The experimental procedure was carried out according to the manufacturer’s instructions. The cell lysate was used as a positive control to test the functionality of the antibodies. 1 to 2 µg total protein was added from cell lysate or EVs to the capillary cartridges (12–230 kDa Wes Separation Module 8 × 13 capillary cartridges, Cat#SM-W002 and 2–40 kDa Wes Separation Module 8 × 13 capillary cartridges, Cat#SM‐W009) to each capillary. Using the Wes system, proteins that correspond with the primary antibodies of CD9 (1:50, Cat#10626D, Thermo Fisher, USA), Flotillin-1 (1:50, Cat#18,634, Cell Signaling, USA), GM-130 (1:50, Cat#12,480, Cell Signaling, USA), Alix (1:50, Cat#2171, Cell Signaling, USA), Annexin V (1:50, Cat#8555, Cell Signaling, USA) were detected automatically. Secondary antibodies were Anti-Rabbit IgG, HRP-linked (1:1000, Cat#7074) and Anti-Mouse IgG, HRP-linked (1:1000, Cat#7076, Cell Signaling, USA). Analysis of protein expression was based on the gel‐like images produced by the Compass for SW software (Version 4.0, Protein Simple).

### Bead-assisted flow Cytometry Analysis of EVs

Surface marker proteins of EVs were characterized using the bead-assisted flow cytometry technique [22]. EVs were bound to aldehyde/sulfate latex beads (4% w/v, 4µM, ThermoFisher, A37304) to allow for conventional flow cytometry analysis. EVs were incubated at RT for 15 min on a shaker with the aldehyde/sulfate beads for adherence. Beads were then mixed into 200µL of PBS containing 2%BSA and incubated for 30 min at 4 °C to prevent non-specific antibody binding. Subsequently, glycine (Merck) was added to a concentration of 100 mM, and samples were incubated for a further 30 min at RT on a shaker. An additional 800µL of PBS was then added, and samples were centrifuged at 2700 g for 3 min to collect EVs carrying beads into a pellet. Beads were then resuspended in 500µL of 1X PBS, and aliquoted into test tubes to be incubated with antibodies for CD9 (Biolegend, 124,808), CD63 (Biolegend 143,904), CD81 (Biolegend, 349,506), HSP70 (Biolegend, 648,004), CD44 (Biolegend, 51-9007233), CD56 (Biolegend, 304,606), SOX-2 (Biolegend, 561,506), Nestin (Biolegend, 560,393), Doublecortin (Biolegend, 561,505) at a 1:100 dilution overnight. After incubation, the beads were washed twice with PBS and the flow cytometry analysis was performed with a BD FACSCalibur Flow Cytometer instrument.

### Cellular Uptake Analysis of EVs

Differential uptake of HDFn-EVs and eND-EVs were analyzed by using confocal microscopy with CMFDA staining [23]. SH-SY5Y cells were grown on sterilized microscope cover slips placed inside 6-well plates. eND-EVs were stained with CellTracker Green CMFDA dye (C7025). Briefly, 20 µl of sucrose-free eND-EVs were mixed with CMFDA dye at a concentration of 5 μm for 30 min at 37 °C. Excess dye was removed using Exosome Spin Columns (4,484,449, Invitrogen). Lastly, cells were treated with 50 µl of fluorescent-labeled eND-EVs for 6 h and analyzed with LS300 (Zeiss).

### Cell Viability Analysis

The effect of eND-EVs on cell viability was determined by MTT assay. Accordingly, SH-SY5Y cells were seeded in 96-well plates at a density of 5000 cells/well. After 24 h of incubation, the cells were treated with 50 µl eND-EVs for 24 and 48 h. Following the incubation period, 10 µl of MTT (Sigma, Munich, Germany) solution (10 mg/ml PBS) was added to each well, and at the end of the three-hour incubation, the medium was discarded and 100 µl of solvent (0.04 N HCl acid/2-propanol) was added to each well. Then, optical density was measured by ELISA plate reader at 540 nm.

### Proteomic Analysis

Proteomic analysis of the samples was performed with tandem mass spectroscopy. Gene ontology enrichment profiles of the eND-EVs proteome were analyzed using Protein analysis through evolutionary relationships (PANTHER) [24]. For PANTHER, the *Homo sapiens* reference database was used to calculate the fold enrichment levels, and the Bonferroni correction was used to correct the p-values. The clipped-GO subset of neural and immune gene ontologies was obtained from a previous study [25]. Redundant GO terms were also filtered out using reduced visualize gene ontology (REVIGO) [26], STRING [27], and Cytoscape Ver.3.9.1 software [28].

### Statistical Analysis

Statistical analysis of the experimental data was done using Graphpad Prism7 software. One-way ANOVA and Two-tailed T test were used for the determination of statistical significance between multiple experimental groups. In the study, T test used for supporting analysis of sample group comparisons involving two factors or condition. On the other hand, ANOVA extended this approach to compare the three or more groups. Therefore, by selecting these statistical tests, it was aimed to strengthen the accuracy of the results obtained from the experiments. *p* < 0.05, < 0.01, < 0.001 were accepted as significantly different and expressed with *, **, *** signs, respectively. All experiments were conducted in three biological replicates.

## Results

### Neurogenic Differentiated Cells Exhibit an Early Neural Characteristic

**Fig. 1:**
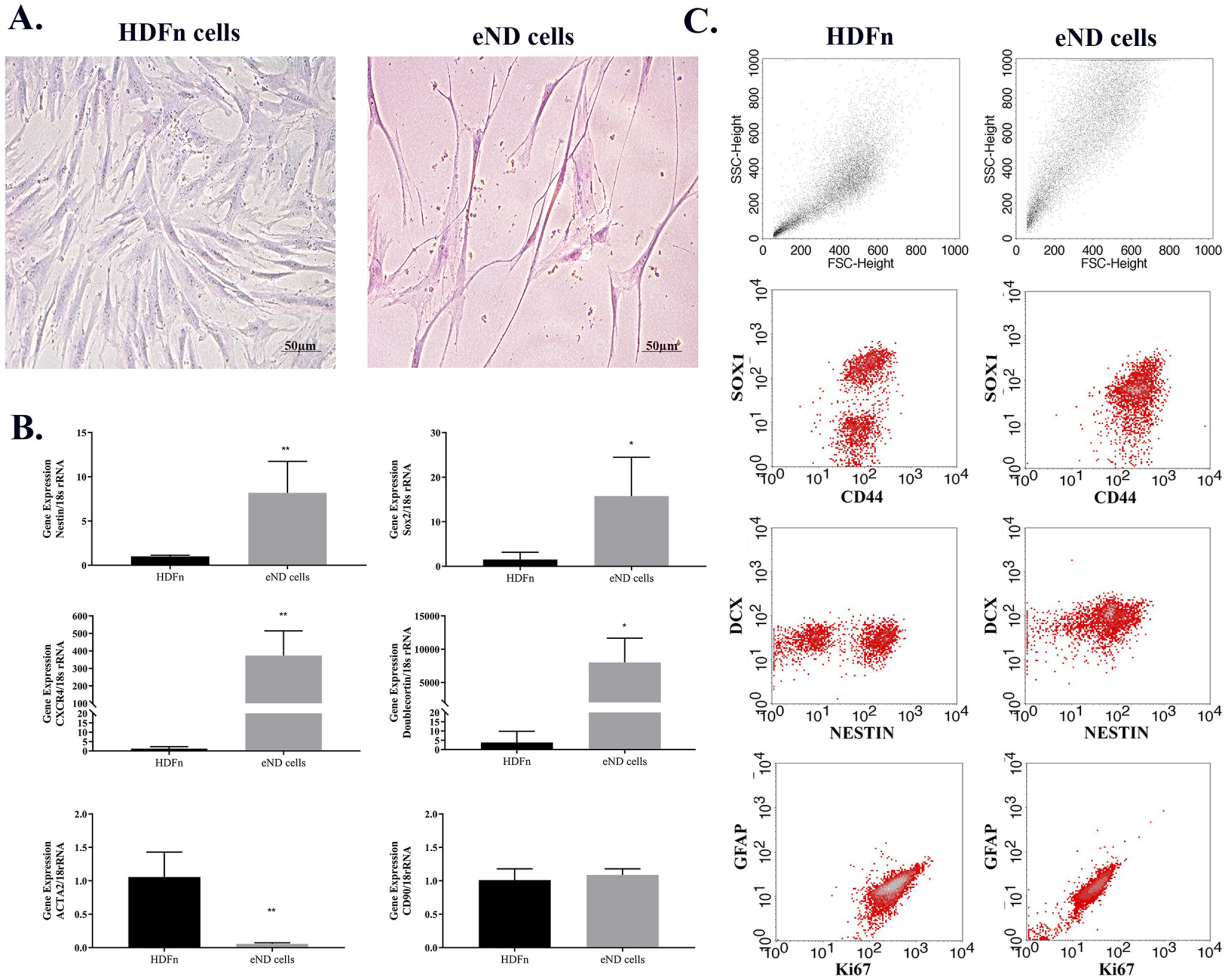
Characterization of early neural differentiated cells. A schematic representation of the early progenitor neural differentiation of HDFn cells (**A**). The nissl bodies in early progenitor neuron differentiated cells (**B**) were examined by cresyl violet staining (scale bar = 50 μm). Changes in gene expression were analyzed by RT-PCR (**C**), while changes in protein expressions were analyzed by flow cytometry (**D**). The statistical analyses were performed using two-tailed T test and all values were expressed as means ± SEM (*n* = 3). *p < 0.05, **p < 0.01

HDFn cells were neurogenically differentiated to modify their EVs with neurogenic characteristics. Differentiated cells were subjected to a set of characterization experiments to determine whether or not they exhibited neuron-like characteristics, as shown in Fig. 1A. Cresyl violet staining showed that the differentiated cells acquired a deep violet color, and their elongated morphology resembled mature neurons (Fig. 1B).

Whether the early progenitor neural differentiated cells expressed neural marker proteins, flow cytometry analysis was performed (Fig. 1C). Compared to their pre-neural differentiation state, the expression of neural progenitor gene SOX1, Doublecortin, and Nestin were increased in differentiated cells. Expression of the proliferation-related protein Ki67 was reduced in neurogenically differentiated cells, while the expressions of CD44 and GFAP were not significantly changed by the differentiation.

Finally, the expression levels of neural genes were compared between differentiated and undifferentiated cells (Fig. 1D). Expressions of Doublecortin, Nestin, SOX2, and CXCR4 were significantly higher in differentiated cells when compared to the undifferentiated control group. Levels of Nestin and SOX2 were increased 8 and 15 times, respectively, while Doublecortin and CXCR4 levels increased more than 500 fold. On the other hand, while the ACTA2 gene as a mesodermal lineage marker was observed to decrease about 20 folds in the early neural differentiated cells, and no alteration was observed in the CD90 gene expression.

### Both HDFn-EVs and eND-EVs Demonstrate Characteristic Features of EVs

**Fig. 2:**
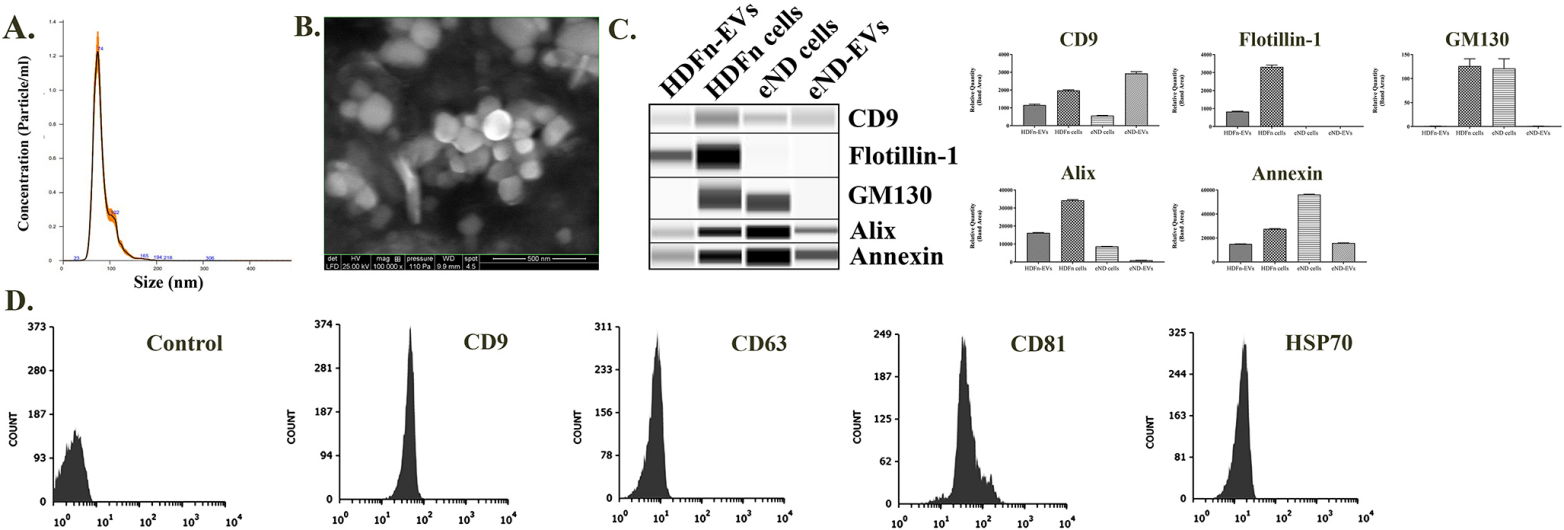
Characterization of HDFn -EVs and eND-EVs. The dimensional features and nanoparticle quantity of EVs (**A**) were performed by the NTA method. To validate size distribution findings, an electron microscopy analysis of EVs (**B**) was performed by environmental SEM (scale bar = 500 nm). Analysis of important protein markers in the EVs was examined by SimpleWestern analysis (**C**) and bead-assisted flow cytometry analysis (**D**). Values are representative of three independent experiments (*n* = 3)

Isolated EVs were characterized with a variety of physical and biochemical characterization methods, as shown in Fig. 2. The quantification and size distribution analysis of the EVs were determined by the NTA method (Fig. 2A). Measurements of the EVs resulted in a non-polydisperse population between 50 and 120 nm, peaking at 74 nm. The concentration of EVs was measured as 3.66 × 10^9^ particles/ml. In the SEM micrographs, EVs appeared as 100 nm spherical particles in SEM images (Fig. 2B). In capillary western blot results, it was shown that EV markers such as CD9, Alix, and Annexin V were positive in both HDFn-EVs and eND-EV groups (Fig. 2C). Moreover, GM130 as a negative marker was positive in cells, while it was negative in EVs as expected. Surprisingly, the Flotillin-1 marker was positive in the HDFn-EV group, yet no signal was observed in the eND-EV group. EVs isolated from early progenitor neural differentiated cells were characterized with bead-assisted flow cytometry to complete their molecular characterization (Fig. 2D). Transmembrane proteins CD9, CD63, and CD81 are commonly associated with EVs, and their presence confirms the presence of the lipid bilayer membrane of the EV, while cytosolic protein HSP70 was used to confirm that this lipid-bilayer enclosed an intracellular cargo. eND-EVs were shown to be positive in all four marker proteins.

### eND-EVs Acquire Molecular Features of Neural Progenitor Cells

**Fig. 3:**
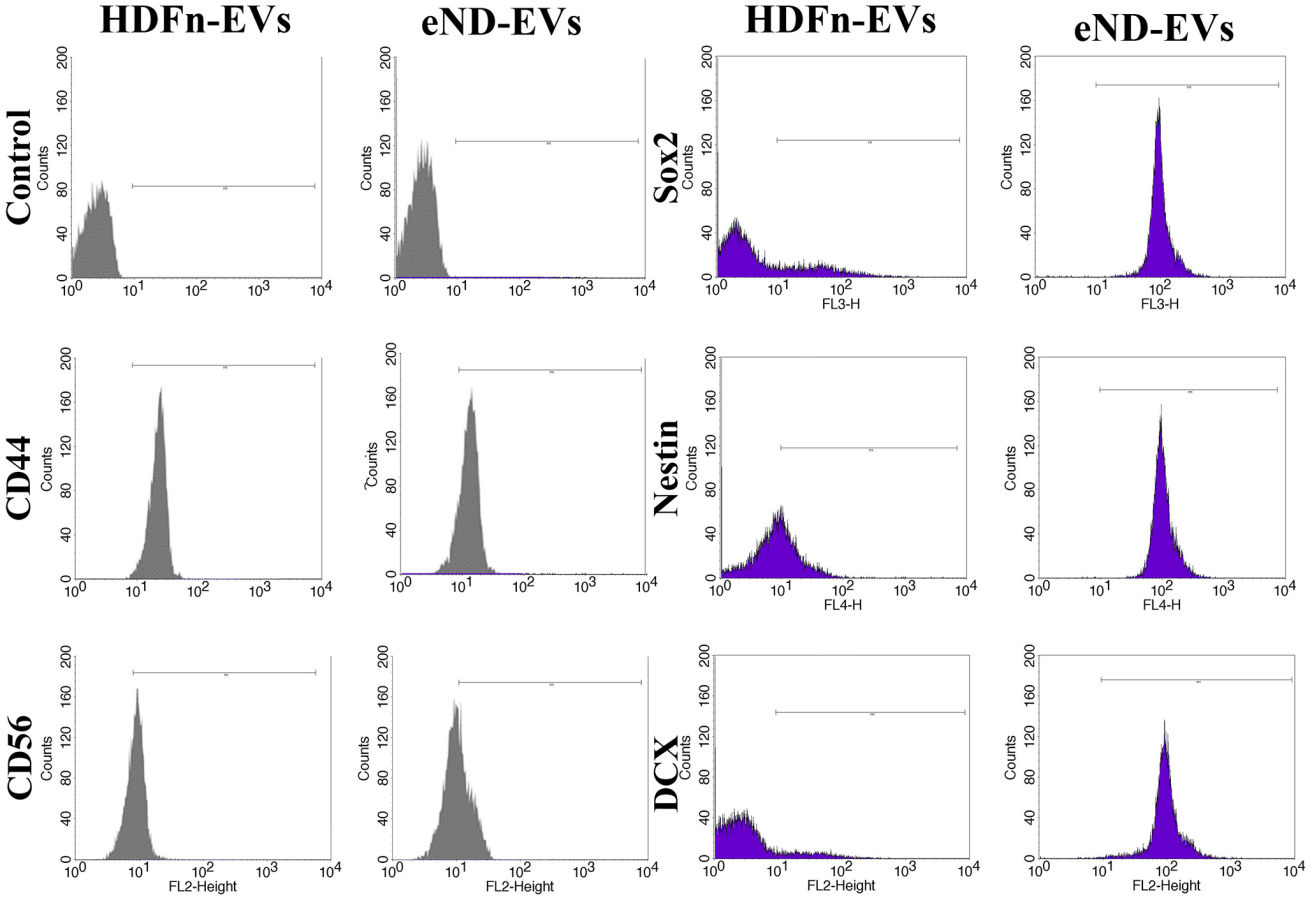
The emergence of neural markers in the eND-EVs of early progenitor neural differentiated cells. Whether EVs in differentiated cells carry neural-associated proteins was observed by flow cytometry analysis. In the analysis, the alterations in the proteins carried by eND-EVs were compared with basal HDFn -EVs. Values are representative of three independent experiments (*n* = 3)

To show that the EVs produced from differentiated HDFn cells had neural characteristics, eND-EVs were subjected to flow cytometry, as shown in Fig. 3. Results showed a considerable decrease in the CD44 expressions in the eND-EVs. Nonetheless, the differentiation procedure increased the expression of neural progenitor markers such as CD56, Sox2, Nestin, and Doublecortin in the eND-EVs.

### eND-EVs are Abundantly Uptaken by Neuroblastoma Cells

**Fig. 4:**
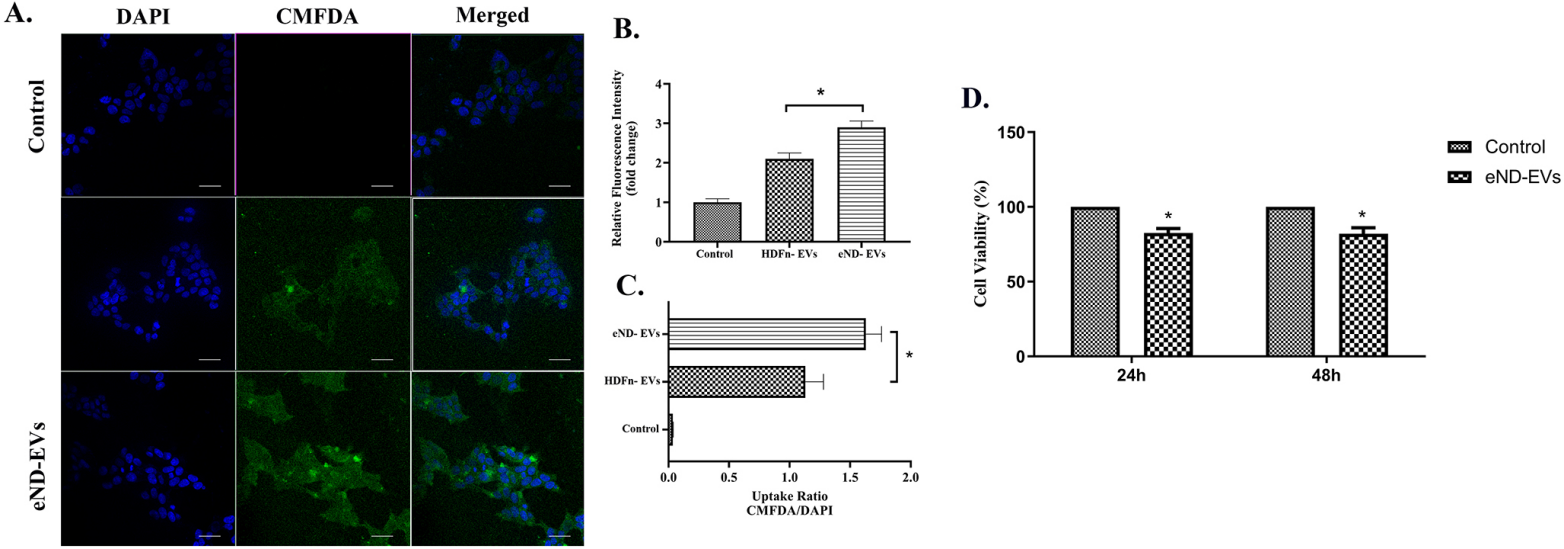
Cell-specific internalization of eND-EVs. The cellular uptake of CMFDA-labeled EVs in both groups was visualized by fluorescence microscopic analysis after 6-hour incubation (20x magnification) (**A**). By measuring the CMFDA signals and normalizing with DAPI, it was observed that the eND-EVs were taken up by the neural SH-SY5Y cells more selectively (**B**, **C**). The effects of eND-EVs on the viability of SH-SY5Y cells at 24 and 48 h (**D**). All values were expressed as means ± SD (*n* = 3). *p < 0.05

To visualize the selective uptake of eND-EVs by neural SH-SY5Y cells, a cellular uptake assay was performed, as shown in Fig. 4. According to the results, it was observed that both eND-EVs and HDFn-EVs were able to enter into the cells successfully (Fig. 4A). Moreover, while the total RFU values in cells increased approximately three times in the eND-EV group compared to the control, the approximately 2-fold increase was observed in the HDFn-EV group (Fig. 4B). However, by measuring the CMFDA signal normalized to DAPI, it was determined that eND-EVs were selectively uptaken by the neural SH-SY5Y, which cellular uptake of eND-EVs was 1.6 times higher than the HDFn-EVs (Fig. 4C).

### eND-EVs Treatment Decrease SH-SY5Y cell Viability

Cell viability assay was performed to determine the effect of eND-EVs on SH-SY5Y cells. eND-EVs were applied to cells for 24 and 48 h. eND-EVs were significantly decreased the viability of cells by ⁓7.5% and ⁓8%, respectively at 24 and 48 h compared to control (Fig. 4D).

### The Proteomics of eND-EVs Display neural-related gene Ontology Terms

**Fig. 5:**
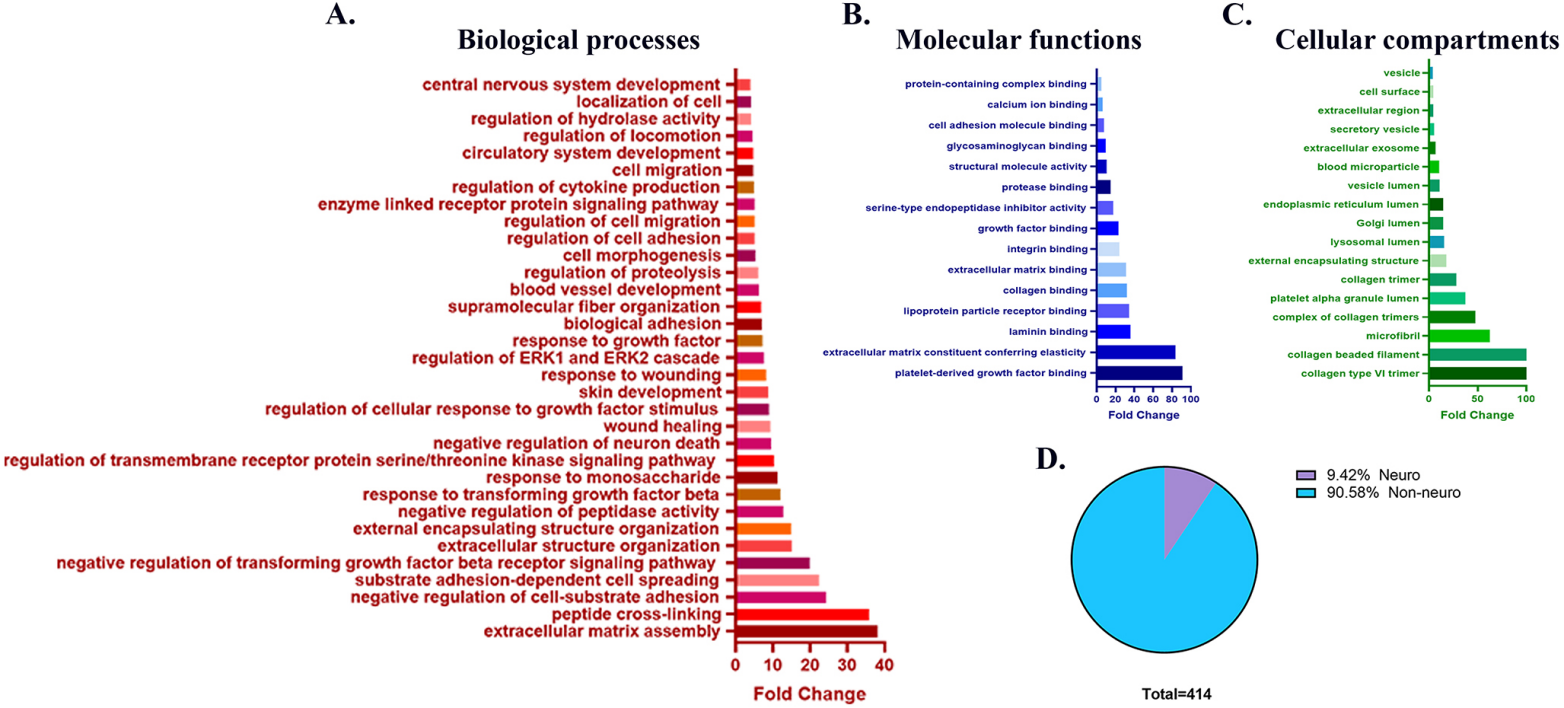
Gene ontology analysis of eND-EVs proteomics. Gene ontology analysis of the identified proteins from eND-EVs in (**A**) biological processes, (**B**) molecular functions and (**C**) cellular components. (**D**) Percentage of the GO terms that were listed in the neural-immune GO ontology subset

Gene ontology enrichment analyses were used to determine the functional enrichment of the eND-EVs proteome, as shown in Fig. 5. The highest degrees of enrichment was observed in the GO terms “extracellular matrix assembly”, “platelet-derived growth factor binding”, and “collagen type VI trimmer” (Fig. 5A, B, and C). Besides, it was observed that 9.42% of the total 414 proteins were neural-related proteins (Fig. 5D). A curated, clipped GO subset focusing on proteins related to the neural and immune systems was used to filter out unrelated terms. In total, 82 eND-EV proteins were associated with 315 individual GO biological process terms. 38 of these terms were included in the neural-immune GO subset.

### The Proteomics of eND-EV has the Specialized Neural gene Ontology Pattern

**Fig. 6:**
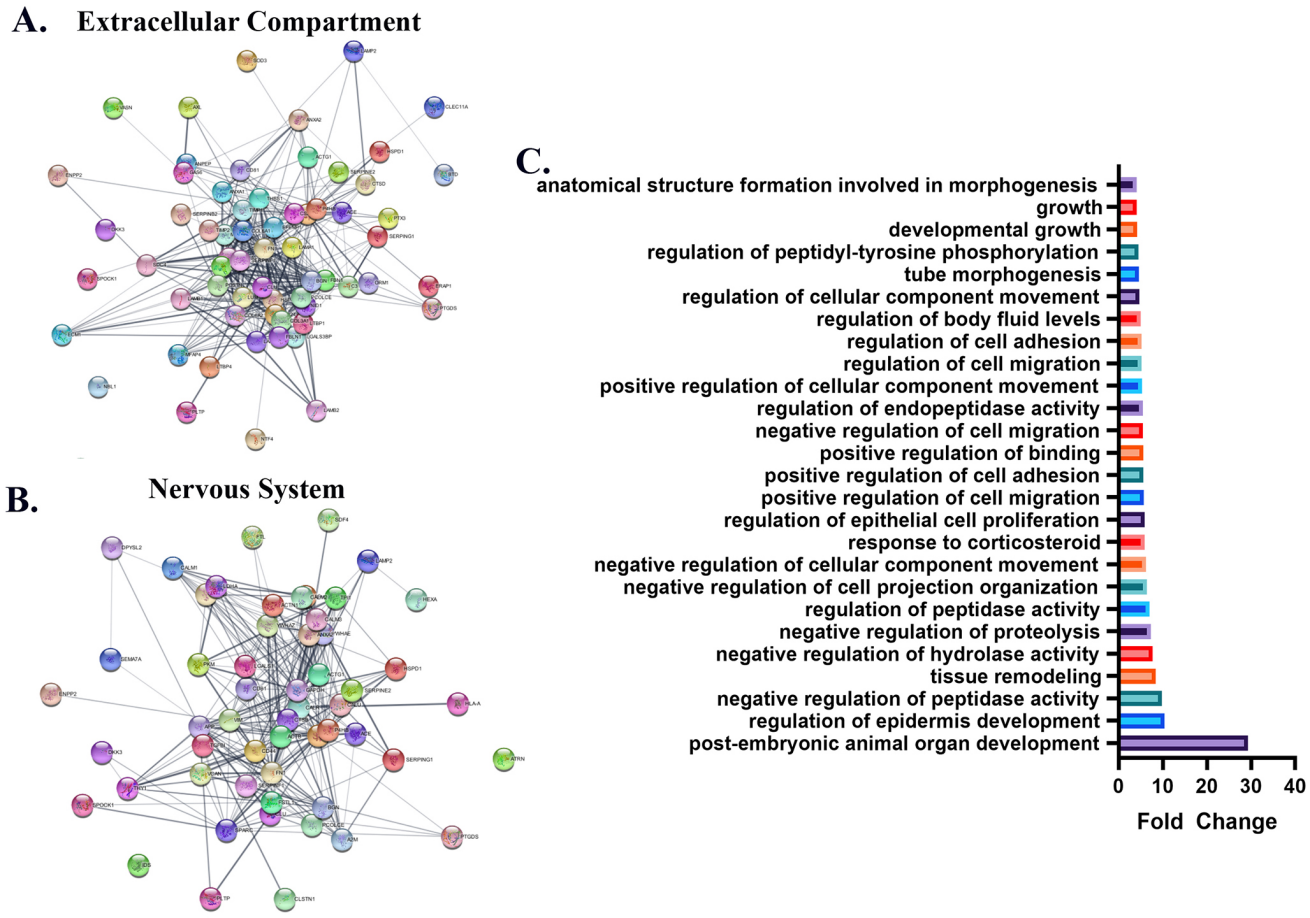
Specialized neurogenic gene ontology pattern of eND-EVs proteomics. Gene network representation of interactions between gene target predictions for (**A**) extracellular compartment and (**B**) nervous system. (**C**) Fold change of GO terms under the neural-immune GO subset compared to the human reference GO database

To examine the proteomic results analyzed in the string database in more detail, using the Cytoscape Ver.3.9.1 software, cellular compartment filters were used to map the proteins specific to that compartment. Protein relationships seen in the map formed by the ‘Extracellular Compartment’ filter of EVs-derived proteins are shown in Fig. 6A. In addition, the ‘Nervous System’ filter was used to show a neurogenic characteristic of these EVs, and the resulting map is shown in Fig. 6B.

## Discussion

The functions and properties of an EV greatly depend on its cell of origin. As neurons are classically unable to proliferate under in vitro conditions, an alternative source of neural EVs may have essential implications in EV research and clinical applications. In this study, we have explored the use of early-neural progenitor cells as an alternative to mature neurons for producing EVs with neural properties, dubbed eND-EVs. They contain vital neural proteins and are preferentially uptaken by neuron-like cells over non-neural EVs. Our findings suggest early-neural progenitor cells can be used for the long-term production of eND-EVs, which may be a source of EVs as delivery vehicles for delivering therapeutics to neurons. Neural characteristics that the cells acquire after their differentiation to neural progenitors are the decisive factor in that drug carriers will be used to target neural tissues. In the literature, it was already demonstrated that HDFn cells can differentiate into neural progenitors [29]. Moreover, the robustness and stability of dermal fibroblasts make them an attractive candidate for long-term culture, expansion and good alternative to multipotent stem cells. Therefore, all these offer many advantages to using fibroblast as a cell source in the differentiation experiments. According to the results of our study, differentiated cells acquire the characteristics of neural progenitors both genetically and morphologically. The morphology of the differentiated cells indicates that cellular transition from the typical spindle-shaped HDFn cells to a specific phenotype of neural cells in which branched and long neurite-like projections neurites or perikaryon-like structures are observed [30]. Moreover, the increase of cresyl violet staining showing the number of ribosomes on the granular endoplasmic reticulum to the levels, which HDFn cells cannot have, supports the transition of differentiated cells to neural characteristics [31]. Around 20 to 25% of the HDFn cells kept their fibroblastic morphology post-differentiation, suggesting that the differentiation efficiency of our process was around 75 to 80%.

Expression of biomarker proteins associated with early neuron progenitors was significantly increased post-differentiation. Two of these proteins, Nestin and CD44, are expressed by HDFn cells, at lower levels of expression compared to early neural progenitors. This was reflected in our results, where the differentiation process increased the expression of Nestin around 5-fold, and the percentage of Nestin or CD44 positive cells increased from around 50% to around 100%. Similar to gene and protein expression patterns of neural progenitors, the downregulation in Ki-67 expression and upregulation of essential progenitor markers such as Doublecortin and CXCR4 is another indicator of this cellular transition [32, 33]. The absence of GFAP expression in differentiated cells is a positive finding that the differentiation process does not lead to astrocytic lineage [34]. On the contrary, the upregulated expression of Sox-1 and Sox-2 genes is an important indicator that the differentiated cells have an orientation towards a neural lineage [35]. On the other hand, in the gene expression analysis, the upregulation of ectodermal lineage-related genes is as important as the downregulation of mesodermal lineage-related genes. Also, in the literature, it was determined that mesodermal markers such as ACTA2 decrease in neuronal differentiated cells [36]. Our results also show that early neural differentiated cells go into a neural lineage based on alterations in the relevant gene expressions. Furthermore, the differentiation induced the expression of proteins not normally expressed by HDFn cells but by early neural progenitors, such as CXCR4 and Doublecortin. The absence of CXCR4 in HDFn cells may have caused a fold increase in protein levels in eND-EVs to appear excessive.

The physical characterization of the EVs shows that they are < 200 nm in diameter and have a spherical shape. The isolated EVs carried transmembrane proteins CD9, CD63, and C81, proving the presence of the lipid bilayer. The vesicles also contained HSP70, a cytosolic cargo protein. The presence of cytosolic cargo enclosed within a lipid bilayer confirms the presence of EVs in our samples. Accordingly, it was understood that eND-EVs were compliant with the MISEV2018 guidelines in terms of basic characterization criteria and nomenclature [37]. On the other hand, the differentiation of HDFn cells might lead to changes in the composition of EVs secreted by those cells [38]. Consistent with their parent cells, proteomic analysis of eND-EVs shows an increased expression of Nestin, Sox2, CD56, and Doublecortin. Some of these proteins, whose expression increases in eND-EVs, can also play a role in neuronal homing capability. Studies in the literature have shown that CD56, also known as NCAM-1, takes part in homing to neuronal tissues and migrating through brain parenchyma via hemophilic binding [39]. Besides, it is known that Doublecortin is quite an important protein for neuronal migration as one of the microtubule-associated proteins [40]. Accordingly, these proteins that appear in eND-EVs are thought to provide both neuronal characteristics and increase the homing capabilities. Besides, our results showed that the Flotillin-1 expression was not detected in both differentiated eND cells and eND-EVs. However, studies in the literature have stated that Flotillin-1 expression decreases in neural differentiated cells [41]. Therefore, we think that this is the reason for the lack of antibody signal from the Flotillin-1 marker in both our differentiated eND cells and eND-EVs, consistent with our findings.

An in vitro uptake assay was used to determine whether or not eND-EVs had an enhanced ability to enter neural cells over other EVs. The uptake of eND-EVs was higher compared to HDFn cells. The difference in uptake is likely caused by the neural proteins carried by eND-EVs, which may enhance cellular uptake by neuron-like cells thanks to the membrane proteins of the recipient cells [42]. Considering the mechanisms by which EVs are taken into cells, the primary mechanisms in the literature are membrane fusion, endocytosis and its subtypes such as macropinocytosis and clathrin-mediated endocytosis [43]. Although these mechanisms have both positive and negative aspects compared to each other, the obtained information cannot explain up to now why eND-EVs are selectively taken up by the cells. On the other hand, EVs are crucial to developing EV-mediated drug delivery systems. CMFDA was used over other fluorescent dyes to ensure that our observations were not caused by unbound dye particles. After passing through EV or cell membranes, CMFDA is converted into a cell impermeant form by resident esterase enzymes. This prevents dye leakage and accumulation within micelles formed by cell membrane fragments, as with other lipophilic dyes [44]. In addition, the effects of eND-EVs on cell viability were examined. eND-EVs have been shown to reduce cell viability at 24 and 48 h.

Analysis of the eND-EVs proteomes revealed that they were enriched in proteins associated with cell adhesion and extracellular matrix-related proteins. We determined the percentage of neural-related GO terms using a previously published neural/immune gene ontology subset [25]. As early progenitors are immature, proliferating neural cells, the differentiated cells and eND-EVs they secreted express a select fraction of neural proteins. This was well represented in our GO analysis, where 38 terms from the neural-immune GO subset were observed in our samples. When comparing the number of proteins under these 38 neural-immune GO terms, we observed that the term “post-embryonic animal organ development” was 29.31 times higher compared to the human GO reference list, supporting the neural-progenitor characteristics of the eND-EVs. Adult neural progenitor cells are associated with maintaining and repairing the central nervous system, explaining the presence of such protein markers [45, 46]. Besides, there are studies in the literature on proteomic analysis of neural progenitor cells-derived EVs (NPC-EVs) [47]. In the studies, it was stated that NPC-EVs take part in not only some neural functions but also neural progenitor-related proteins such as Teneurin-4 and Semaphorin-6 A, which are present in the NPC-EVs. Our proteomic results also showed that proteins such as Semaphorin-7 A, Neuropilin-1, and Vinculin, which play a role in neural functions, were determined in the protein content of eND-EVs [48–50]. Moreover, it was determined that the characteristic markers of early neural progenitor cells such as SOX-2, Nestin, and Doublecortin were presented in the eND-EVs. Examining the neural EV studies in the literature, our findings provide novel insights into the acquired neural properties of eND-EVs. In our results, it was found that eND-EVs had neural proteins like Doublecortin, Neuropilin-1, Semaphorin-7a and also eND-EVs were preferentially taken up by neuron-like cells. Therefore, these findings closely align with and expand upon the work of Campero-Romero et al., who showed that NPC-derived EVs carried neural proteins and they are involved in many neural functions [47]. Moreover, the detection of neural proteins in eND-EVs in our results correlates with Gao et al.’s findings that NPC-derived EVs carried neural proteins such as growth factors [51]. Notably, our observation related neural molecules in the eND-EVs parallels with the Takeda et al.’s findings that NPC-derived EVs had neural-derived biomolecules like proteins and small RNAs [52]. Consequently, all these complementary findings confirm and extends prior studies elucidating the neural properties of eND-EVs in neural functions.

The findings of our study offer feasible implications for the future development of targeted-EV based therapies for neural disorders. By showing that eND-EVs have acquired neural features, including preferential uptake by neuron-like cells and carrying neural proteins, our study indicates that may serve as promising carriers for delivering therapeutic cargo to the nervous system. Moreover, the fact that EVs can transfer a various molecule while avoiding rapid clearance could provide a delivery system directly to sites of injury or disease in the nervous system [53]. While the eND-EVs in our study show important potential for drug delivery to neural tissues, there are several limitations that need to be addressed by future studies. Firstly, the neonatal fibroblast cells used for studying the differentiation are male origin. Thus, the possibility that differentiation efficiency, duration and EVs’ content of female origin fibroblast may change under the same experimental conditions should be taken into consideration. Although HDFn cells are promising cell sources for differentiating early neural progenitor cells, there are important ethical implications that need to be considered. The potential use of the eND-EVs will elevated by addressing these issues such as the use of human-derived materials as cell sources and informed consent [54]. Secondly, the stable cargo loading and controlled release of drugs need to be optimized for efficient use of EVs as drug carriers. Moreover, it is likely required a better comprehension of the molecular mechanisms that influence neural targeting, such as receptor interactions, to develop more sophisticated drug delivery tools. We thought that addressing these limitations helps to realize the full potential of EV-based delivery strategies.

In conclusion, it was observed that the neural differentiation of HDFn cells led to a similar differentiation of the molecular properties of EVs. Accordingly, it was proved that eND-EVs acquired neural features and were selectively taken up by neuroblastoma cells compared to undifferentiated HDFn-EVs. Moreover, this study revealed that eND-EVs has potential as a promising drug carrier for the treatment on neuroblastoma.

### Electronic Supplementary Material

Below is the link to the electronic supplementary material.

Supplementary Material 1

Supplementary Material 2

Supplementary Material 3

Supplementary Material 4

Supplementary Material 5

Supplementary Material 6

Supplementary Material 7

## Data Availability

All the proteomic results are available within the paper and its Supplementary Information.
