# Supplementary figures and images for: A Perspective on the Characterization of Early Neural Progenitor Cell-Derived Extracellular Vesicles for Targeted Delivery to Neuroblastoma Cells
Source: Neurochem Res. 2024 Jun 5;49(9):2364–78. doi: 10.1007/s11064-024-04165-1 (PMC11310242; doi:10.1007/s11064-024-04165-1)

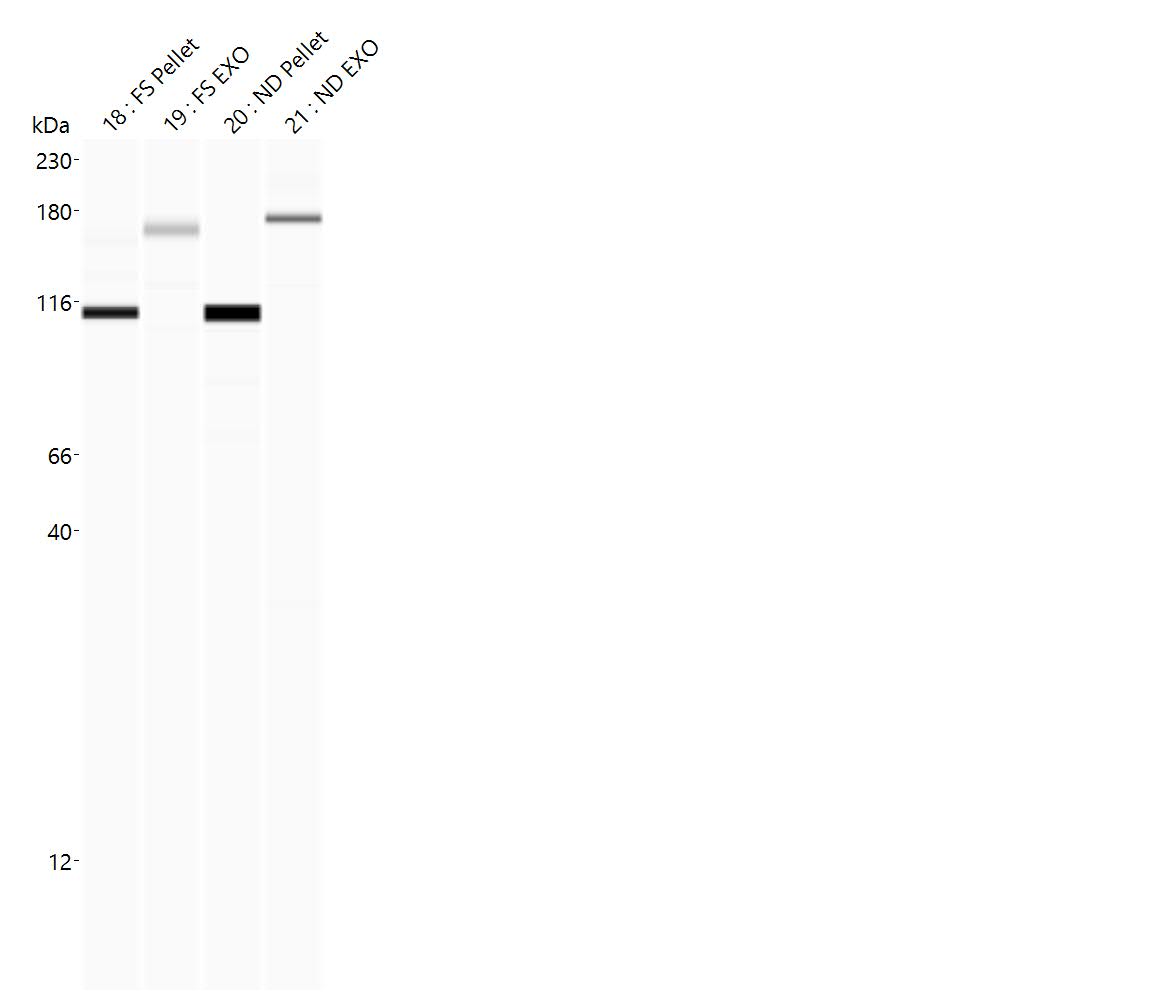

Supplement: Supplementary file 2 — Supplementary Material 2 [file 11064_2024_4165_MOESM2_ESM.jpg]

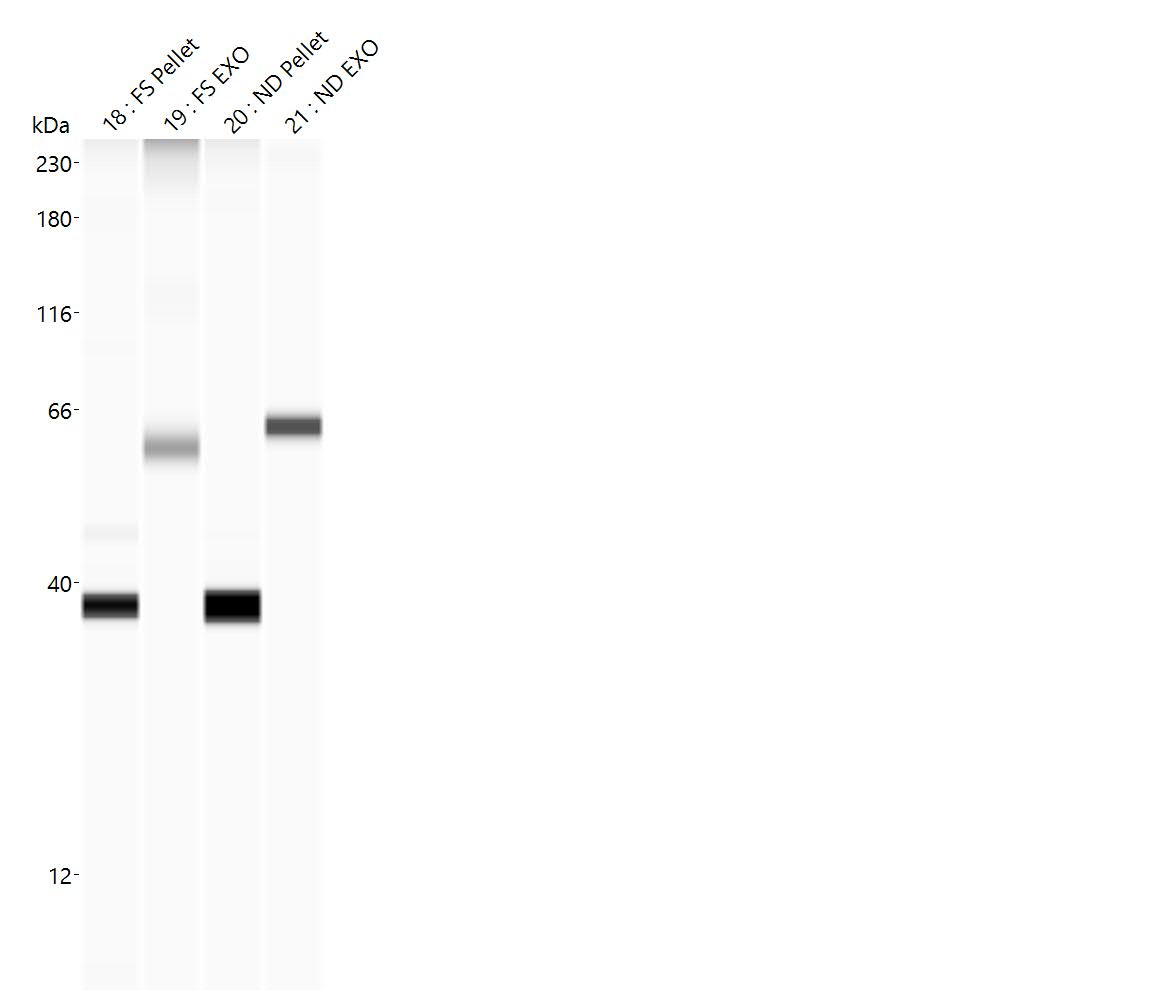

Supplement: Supplementary file 3 — Supplementary Material 3 [file 11064_2024_4165_MOESM3_ESM.jpg]

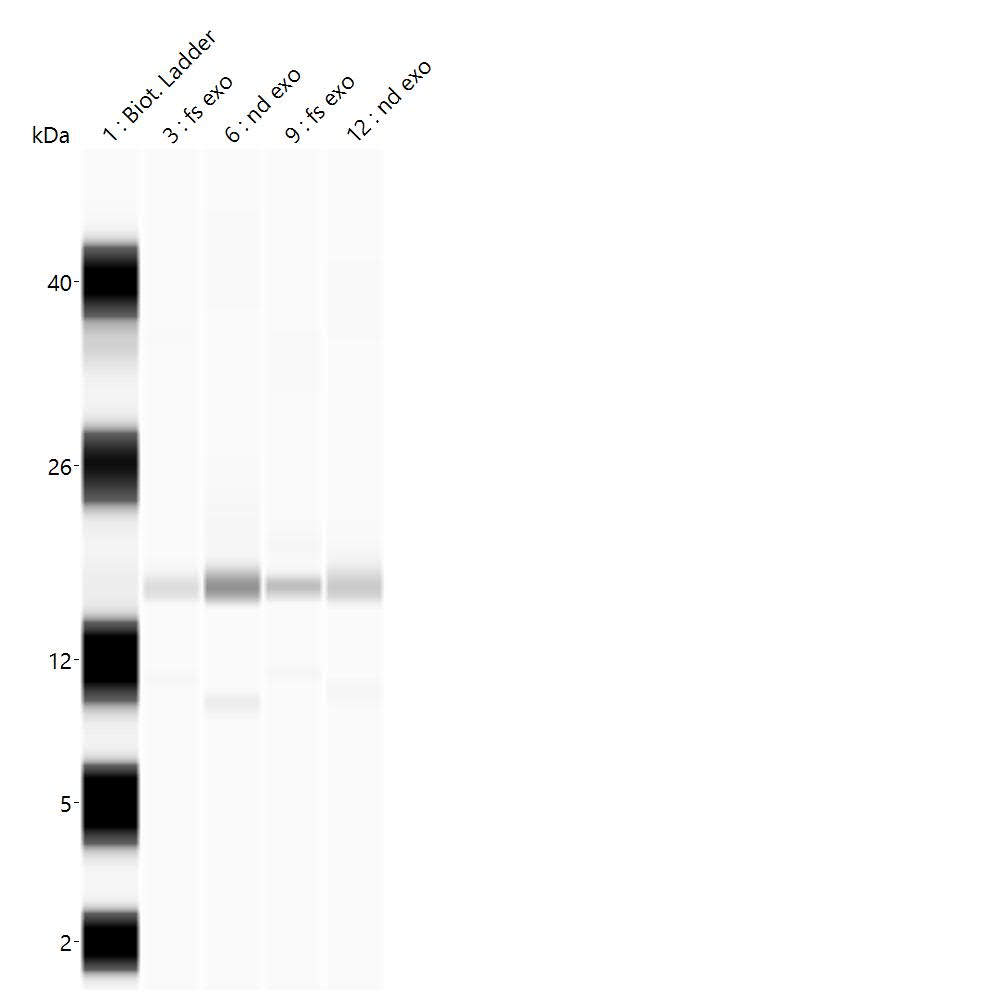

Supplement: Supplementary file 4 — Supplementary Material 4 [file 11064_2024_4165_MOESM4_ESM.jpg]

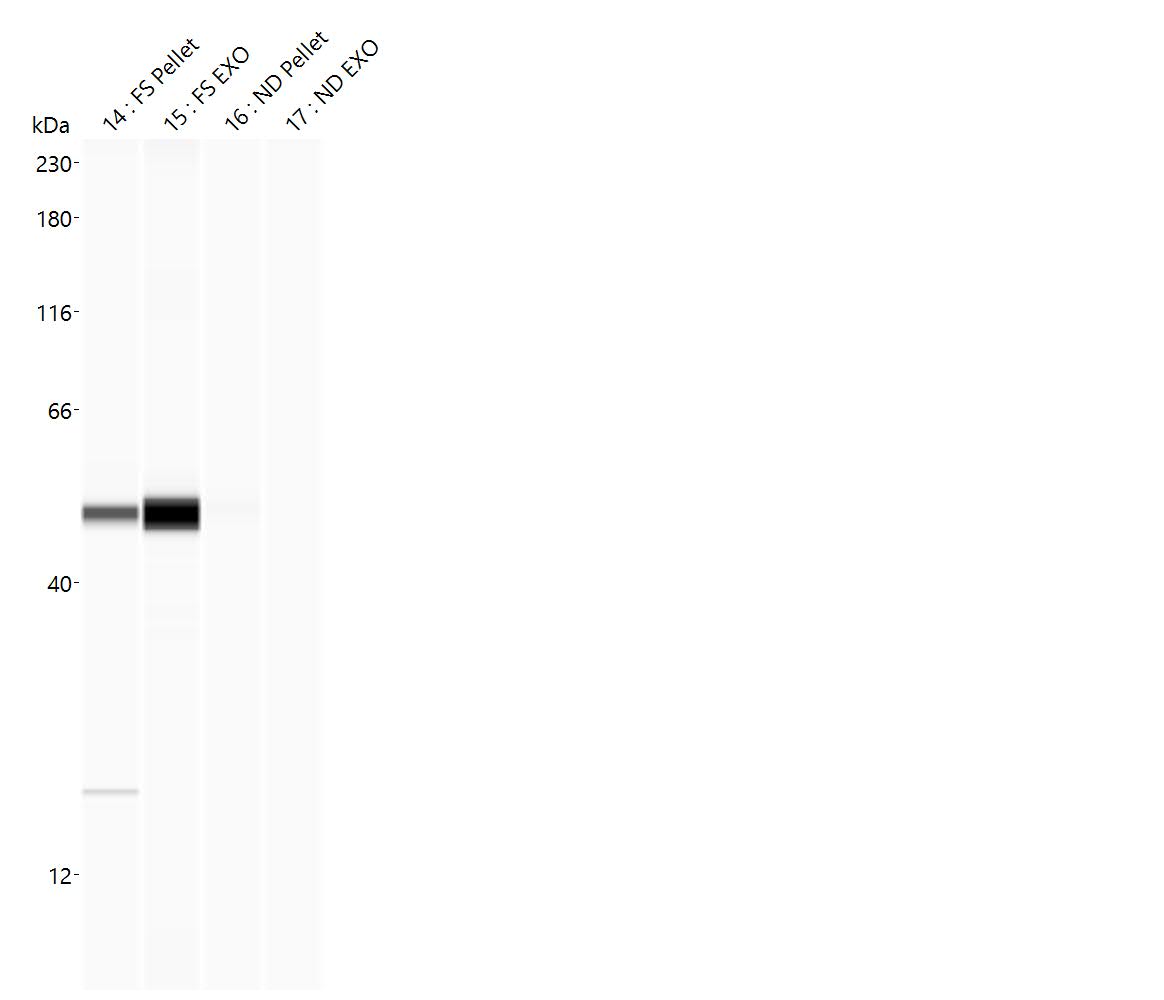

Supplement: Supplementary file 5 — Supplementary Material 5 [file 11064_2024_4165_MOESM5_ESM.jpg]

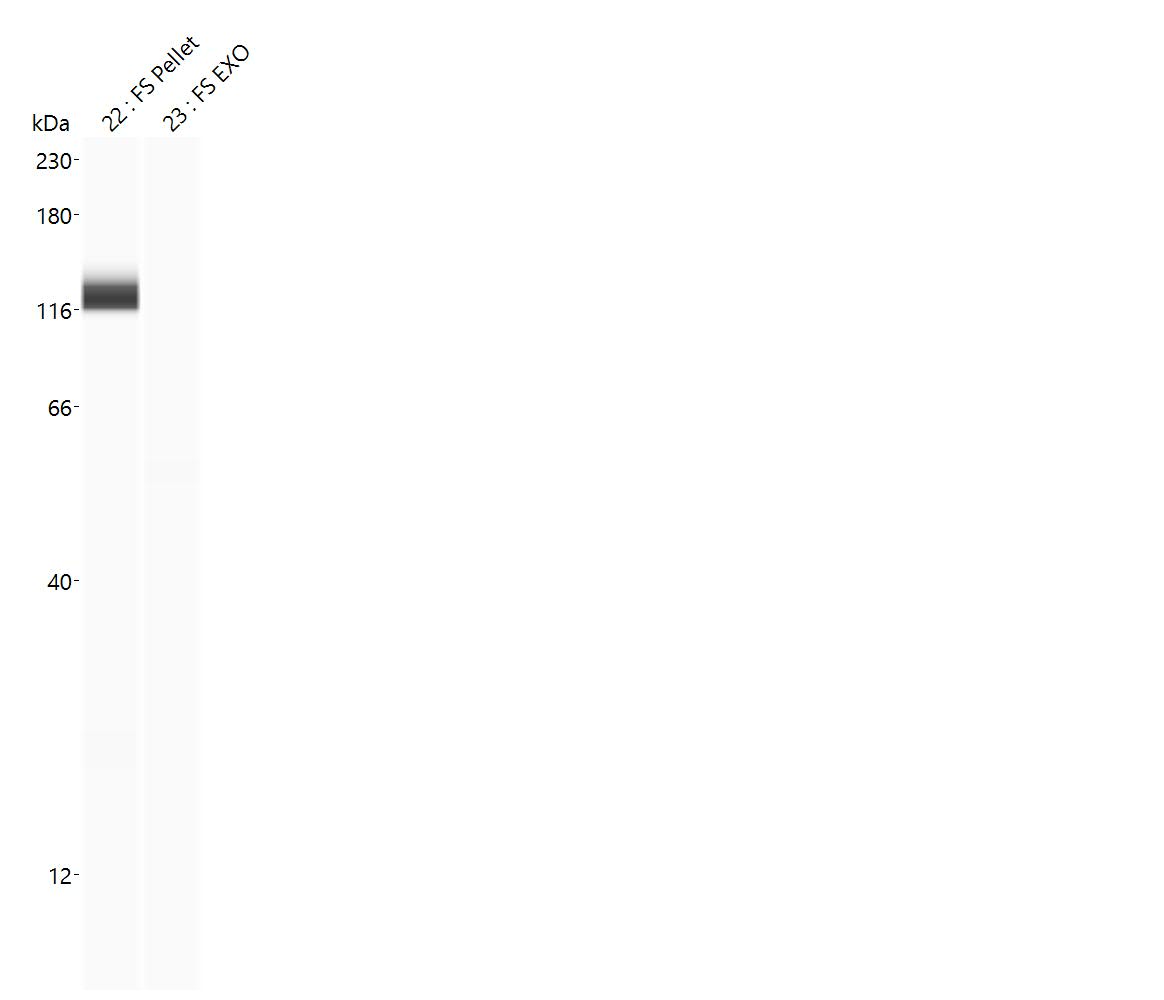

Supplement: Supplementary file 6 — Supplementary Material 6 [file 11064_2024_4165_MOESM6_ESM.jpg]

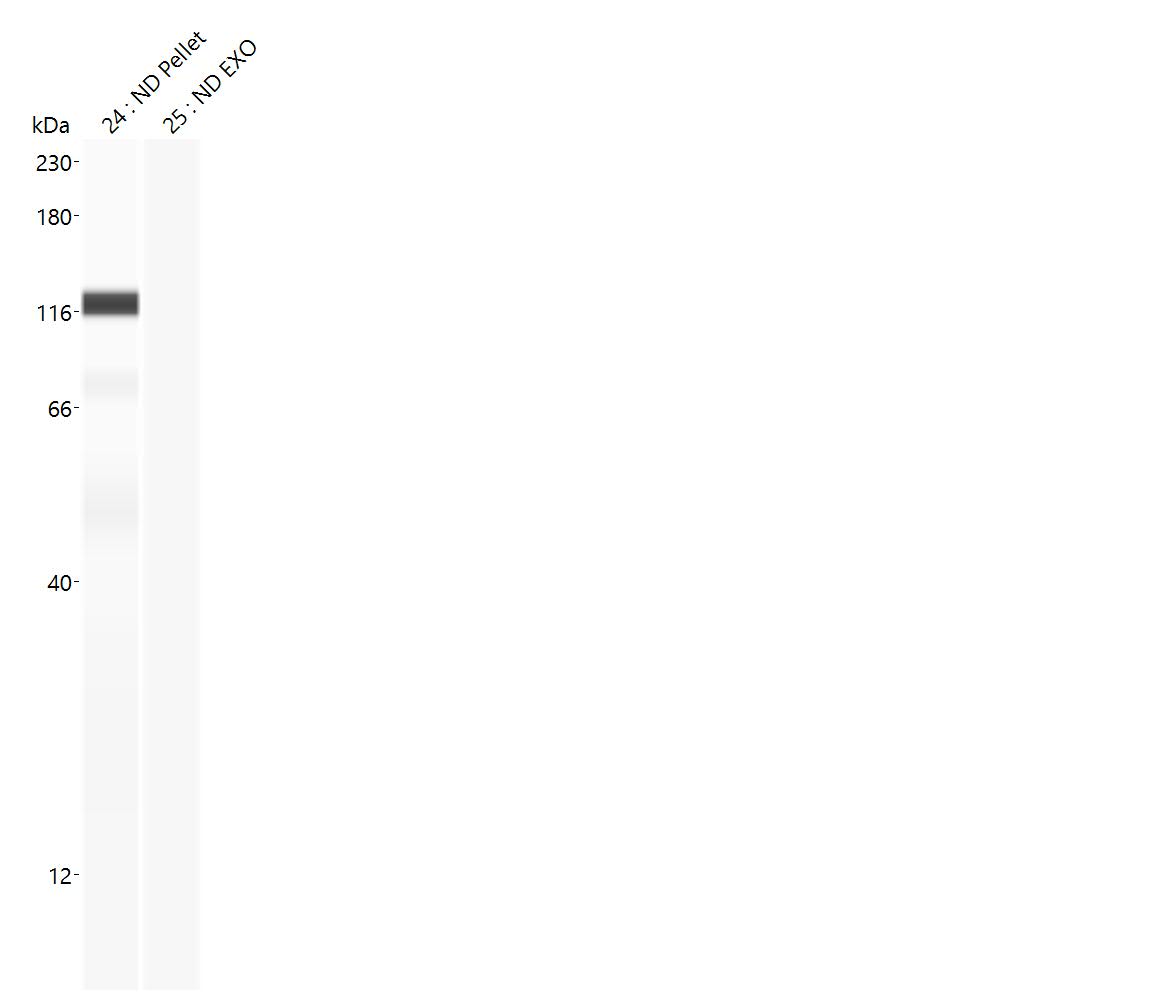

Supplement: Supplementary file 7 — Supplementary Material 7 [file 11064_2024_4165_MOESM7_ESM.jpg]
